# Assessment of a novel biomechanical fracture model for distal radius fractures

**DOI:** 10.1186/1471-2474-13-252

**Published:** 2012-12-18

**Authors:** Sebastian Felix Baumbach, Enrico Dall’Ara, Patrick Weninger, Anna Antoni, Hannes Traxler, Martin Dörr, Philippe K Zysset

**Affiliations:** 1 Department of Surgery, Experimental Surgery and Regenerative Medicine, Ludwig-Maximilians-University Munich, Munich, Germany; 2 Institute of Lightweight Design and Structural Biomechanics, Vienna University of Technology, Vienna, Austria; 3 Lorenz Boehler Trauma Hospital, Ludwig Boltzmann Institute for Experimental and Clinical Traumatology, Cluster for Tissue Regeneration, Vienna, Austria; 4 Center of Anatomy and Cell Biology,Department of Systematic Anatomy, Medical University Vienna, Vienna, Austria; 5 Munich Cancer Registry; IBE / Clinic Großhadern, Ludwig-Maximilians University Munich, Munich, Germany; 6 Institute of Surgical Technology and Biomechanics, University of Bern, Bern, Switzerland

**Keywords:** Biomechanical fracture model, Distal radius fracture, Fresh-frozen, Plate osteosynthesis

## Abstract

**Background:**

Distal radius fractures (DRF) are one of the most common fractures and often need surgical treatment, which has been validated through biomechanical tests. Currently a number of different fracture models are used, none of which resemble the in vivo fracture location. The aim of the study was to develop a new standardized fracture model for DRF (AO-23.A3) and compare its biomechanical behavior to the current gold standard.

**Methods:**

Variable angle locking volar plates (ADAPTIVE, Medartis) were mounted on 10 pairs of fresh-frozen radii. The osteotomy location was alternated within each pair (New: 10 mm wedge 8 mm / 12 mm proximal to the dorsal / volar apex of the articular surface; Gold standard: 10 mm wedge 20 mm proximal to the articular surface). Each specimen was tested in cyclic axial compression (increasing load by 100 N per cycle) until failure or −3 mm displacement. Parameters assessed were stiffness, displacement and dissipated work calculated for each cycle and ultimate load. Significance was tested using a linear mixed model and Wald test as well as t-tests.

**Results:**

7 female and 3 male pairs of radii aged 74 ± 9 years were tested. In most cases (7/10), the two groups showed similar mechanical behavior at low loads with increasing differences at increasing loads. Overall the novel fracture model showed a significant different biomechanical behavior than the gold standard model (p < 0,001). The average final loads resisted were significantly lower in the novel model (860 N ± 232 N vs. 1250 N ± 341 N; p = 0.001).

**Conclusion:**

The novel biomechanical fracture model for DRF more closely mimics the in vivo fracture site and shows a significantly different biomechanical behavior with increasing loads when compared to the current gold standard.

## Background

Distal radius fractures (DRF) are one of the most common fractures in adults. The current operative gold standard for the treatment of DRF is volar plating [1,2], aiming at restoring joint integrity and physiological angles with early mobilization of the wrist after surgery [3]. Biomechanical fracture models are used to design, test, and validate devices for osteosynthesis. Today, the standard setup for biomechanical fracture models of dorsally unstable DRF is a dorsal wedge osteotomy 20 mm proximal to the articular surface [4-9]. Few other studies used different osteotomy locations, i.e. 10 to 25 mm proximal to the articular surface [10-13], at the distal border [14], proximal to the sigmoid notch [15], or 3 mm proximal to the distal radio-ulnar joint [16]. No validation of the biomechanical fracture models (i.e. justification of the osteotomy location) could be found for any model in literature.

**Figure 1:**
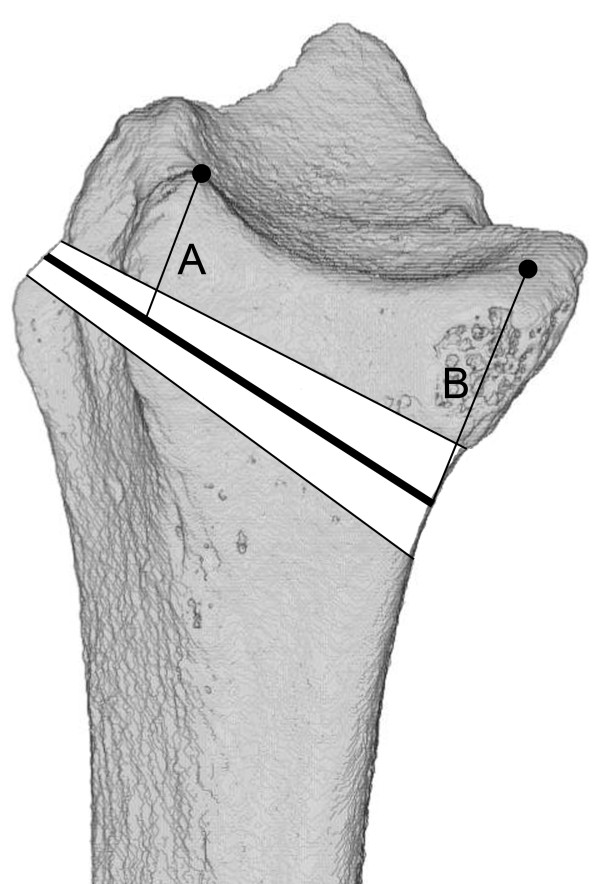
**Distal fracture line location for Colles**’ **fractures. A**) 7.9 ± 2.7 mm; **B**) 11.7 ± 3.9 mm. Adapted from Baumbach et al. [19].

In Europe, dorsally displaced distal radius fractures are commonly called distal radius fractures *loco typico*, referring to the distal 10% of the distal radius [17,18]. A recent study [19] was the first to systematically investigate the exact location of the distal fracture line (DFL). DFL location was found to be well-defined, dorsal 7.9 ± 2.7 mm and palmar 11.7 ± 3.9 mm proximal to the dorsal/palmar apex of the lunate fossa, running oblique from palmar proximal to dorsal distal (Figure 1). DFL location was found to be independent of fracture complexity, energy of the fall, and age. This location challenges the reliability of today´s biomechanical fracture models.

With no reliable biomechanical fracture model available and based on the novel definition of the in vivo fracture location of Colles’ fractures, the authors developed a new biomechanical fracture model for dorsally displaced distal radius fractures. This is illustrated in Figure 2.2.

**Figure 2:**
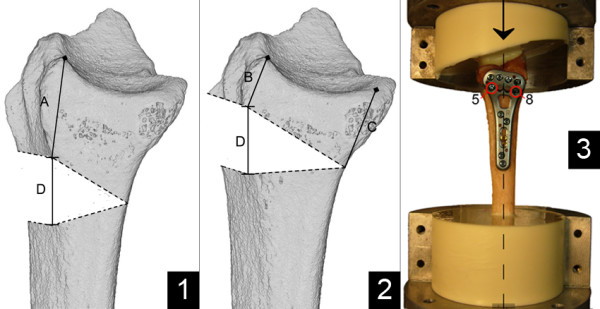
**Illustration of different osteotomy locations and the final setup. 1**) Gold-standard osteotomy location: **A**: 20 mm; **D**: 10 mm; **2**) Novel osteotomy location: **B**: 8 mm; **C**: 12 mm; **D**: 10 mm; **3**) Final biomechanical setup: Arrows and dashed line mark axis of force, screws numbered 5 and 8 were placed following cutting of the osteotomy.

In a preliminary study, the novel biomechanical fracture model was compared to the current gold standard using 14 synthetic radii (large left radius #1027, Sawbone, Sweden). The biomechanical setup was similar, the loading protocol differed to that presented in the current study. The samples were loaded in axial compression with ten cycles in the elastic range followed by compression until 5 mm displacement and consecutive unloading. None of the assessed mechanical parameters (stiffness, dissipated work, load at 5 mm displacement and residual displacement) differed between the two fracture models (Table 1). Interestingly, there was a difference in the type of failure of the construct for high loads. Plate-screw interface failure occurred in only one case for the gold standard (at −1256 N and −3.81 mm displacement) but in four cases of the new biomechanical fracture model (at −1522 N ± 104 N and −3.93 mm ± 0.52 mm displacement).

**Table 1:** Descriptive and comparative statistics for the assessed biomechanical parameters for preliminary study on sawbone

	**Old osteotomy**				**New osteotomy**				
	**Mean**	**SD**	**95% Conf**. **interv**.^**3**^		**Mean**	**SD**	**95% Conf**. **interv**.^**3**^		** T-test**
			**Lower**	**Upper**			**Lower**	**Upper**	
Axial Stiffness [N/mm]	793	129	674	912	742	89	660	825	0.404
Dissipated Work Cycles [N*mm]	2.5	2.6	0.1	4.9	2.8	2.4	0.5	5.0	0.834
Dissipated Work Monotonic [N*mm]	3700	228	3489	3910	3698	302	3418	3978	0.991
Load at −5 mm^1^ [N]	−1623	145	−1756	−1489	−1517	166	−1671	−1363	0.229
Res Displacement^2^ [mm]	−2.18	0.38	−2.53	−1.83	−2.31	0.29	−2.58	−2.04	0.483

^1^Load at −5 mm displacement.

^2^Residual Displacement after unloading.

^3^95% Confidence Interval for Mean.

The aim of this study was to compare the current gold standard biomechanical fracture model for dorsally displaced distal radius fractures and the authors’ novel fracture model using ex vivo cyclic mechanical testing on human fresh frozen radii.

## Methods

### Specimen preparation

Eleven paired, fresh-frozen radii were obtained from the Center of Anatomy and Cell Biology, Medical University of Vienna, AT in compliance with the rules of the local ethics commissions (AUVA 16/2011; LMU 024–12). Prior to testing, bone mineral density (BMD) and bone mineral content (BMC) were assessed at the most distal 10 mm of each radius using a μCT (VivaCT 75, SCANCO Medical AG, Switzerland). Based on the scout views of the μCT, specimens with severe pre-existing osteoarthritis, previous fractures, bone cysts or tumor were excluded.

The specimens were thawed five hours prior to preparation. Variable angle locking volar plates (ADAPTIVE 2.5®, A-4750.61, Medartis Inc., Basel, Switzerland) were mounted following the manufacturer’s recommendations. The manufacture’s drill guide block (A-2723.01) was used to assure uniform placement of the two distal screw rows. The two proximal screws (Figure 2.3, #5,8) were placed after the osteotomy was performed, as they would have otherwise interfered with the cut in the new model. Randomization of the osteotomy locations was achieved by randomly picking pairs of radii and alternating the side for the new osteotomy. The osteotomy was performed using a handsaw and the volar cortex separated completely (1 mm gap, i.e. width of the saw blade). Figure 2 illustrates the gold standard (Figure 2.1) and novel osteotomy location (Figure 2.2). Following the mounting of the plate and cutting of the osteotomy, the specimens were cut 50 mm proximal to the proximal tip of the plate and aligned in a custom-made aluminum jig. Each radius was aligned within the aluminum containers and consequently within the servo-hydraulic material testing system (MTS 858 MiniBionix, MTS Systems Corp., USA), as reported previously [20]. The proximal 40 mm of the shaft were embedded in polyurethane [PUR, FDW Handelsges, Austria]. Distally, a life size negative imprint of the articular surface was created from the same material for each sample. The life size negative imprint ensured sound fitting of the mold to the distal articular surface and did not constrain the distal fracture fragment. The final setup is presented in Figure 2.3.

### Biomechanical setup and statistics

Using the custom-made aluminum jigs, the specimens were mounted in the testing machine. Cyclic, axial compression tests with increasing load were performed. A similar protocol has been used in a previous study on trabecular bone [21]. A preload of −20 N was applied to maintain uniform contact between the life size model and the distal joint surface. The specimens were then tested cyclically, at a constant compression rate of 300 N/s to an initial maximum of −100 N. For each cycle, the maximum load was increased by 100 N until one of the following criteria was met: failure of the construct or 3 mm of displacement.

**Figure 3:**
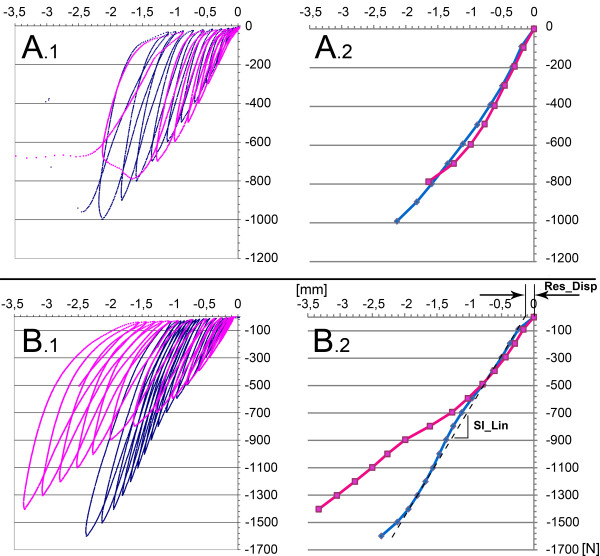
**Exemplary **‘**force **- **displacement**’ **curves (1) and **‘**maximum load**-**displacement**’ **curves. **(**2**). Dark: standard fracture model; Clear: novel fracture model; Top: atypical (n = 3) force-displacement curve (**A1**) and maxima (**A2**); Bottom: typical (n = 7) force-displacement curve (**B1**) and maxima (**B2**). An example of the computation of the residual displacement (Res_Disp) from the linear portion of the 'maximum load-displacement' curve (Sl_Lin) is shown in B2.

Load–displacement curves were recorded and a ‘maximum load-displacement’ curve was computed for each sample from the maximum load of each cycle. To minimize the effect of boundary conditions (i.e. to correct for the differences in the initial, nonlinear portions of the load–displacement curves due to sample variables such as cartilage thickness, etc.), the slope of the linear part of the resulting ‘maximum load-displacement’ (Figure 3B.2: Sl_Lin) curve was computed and the residual displacement (Figure 3B.2: Res_Disp) subtracted from the measured displacement. The following parameters were calculated for each cycle: displacement at the maximum load, dissipated work and the slope between the points at maximum load of two contiguous cycles. For each specimen the total thawed time was kept below 24 h to prevent any alterations in the biomechanical behavior of the bone [22].

For all biomechanical parameters assessed, mean and standard deviations (SD) were calculated. To see whether significant differences exist between the old and new model, a linear mixed model was fitted and tested via a Wald test. Significance for ultimate failure load was tested via t-test. Statistical calculations were performed using the free software R (version 2.11) with a level of significance of 0.05.

## Results

One pair was excluded due to a space-occupying lesion. Ten paired specimens, seven female, with an average age of 74 ± 9 years were tested. Neither BMD (136 ± 55 mgHA/cm^2^ vs. 145 ± 57 mgHA/cm^2^, p = 0.12) nor BMC (581 ± 286 mg vs. 570 ± 274 mg, p = 0.45) varied significantly between the two groups. All specimens were tested successfully.

Exemplary force-displacement and ‘maximum load-displacement’ curves are presented in Figure 3. Within each pair, all force displacement curves showed a similar behavior for lower loads. In seven pairs the gold standard fracture model showed significant stiffening with increasing loads compared to the new fracture model (Figure 3B). In three pairs this stiffening was not present (Figure 3A), with the new osteotomy location failing prior to the standard osteotomy location (New model : Gold Standard; 900 N : 1000 N; 800 N : 1000 N; 900 N : 1800 N). The ultimate load resulted in failure in 11 specimens (New model: n = 5; Gold Standard: n = 6) with the remaining exceeding 3 mm displacement.

**Figure 4:**
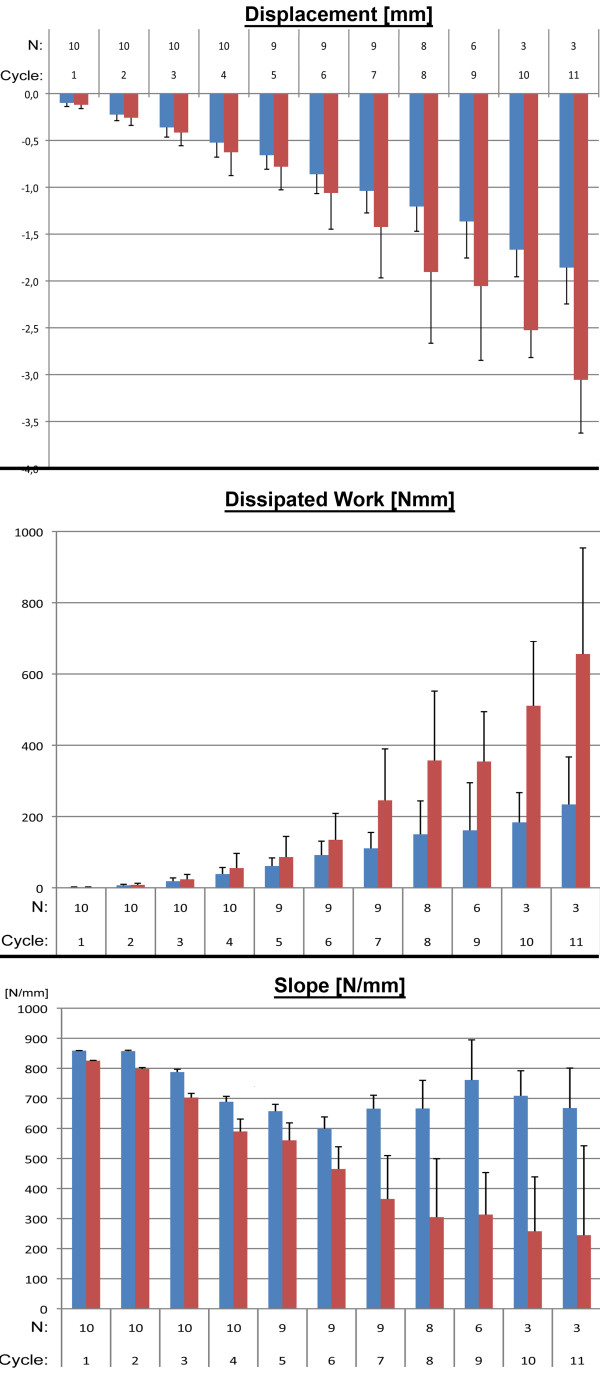
**Mean and standard deviation illustrated for each cycle for displacement**, **dissipated work and stiffness. **Dark: standard model; Clear: novel model; N: number of pairs; Cycles: number of cycles (cycle number times 100 N equals ultimate load for that cycle).

Figure 4 plots the mean and standard deviation for each cycle for displacement, dissipated work and stiffness. The linear mixed model was tested via a Wald test and found the new model to be significant different to the gold standard model (p < 0.001). The average final load resisted, defined as the load of the final cycle completed, were significantly lower (p = 0.001) in the new model (860 N ± 231.9 N) than in the standard model (1250 N ± 340.7 N).

## Discussion

In this study, an improved biomechanical model for dorsally unstable distal radius fractures was developed and tested against the current gold standard model. Both models were compared in ten paired fresh-frozen radii using a standard biomechanical setup. Significant differences were found for displacement, dissipated energy, stiffness, and final loads resisted.

The authors hypothesized that a more distal defect would, on the one hand, change the lever arm and, therefore, the force transmission within the bone-screw-plate construct. This on the other hand was expected to alter the anchorage of the distal screws due to the significantly reduced distal bone stock volume.

As mentioned in the introduction, a preliminary study on synthetic radii was carried out to evaluate the possible geometric influences of the different osteotomy locations. None of the biomechanical parameters assessed differed significantly between the two groups; which is most likely due to the elastic properties of the sawbone.

Conducting the same experiment in fresh-frozen radii resulted in significant differences. As expected, the greater lever arm of the new fracture model resulted in lower stiffness and higher dissipated work values compared to the standard model. The observed difference became more pronounced with increasing loads, which might be explained by the fact that transmission of lower loads is primarily influenced by the position of the plate. This is not until the plate starts to bend that the lever arm (i.e. the osteotomy location) becomes an influential parameter.

No significant difference could be found for screw-bone failure between the two fracture models, despite the different distal bone stock volume; this is in line with recent studies by Greenberg et al. [23] and Weninger et al. [24]. Greenberg et al. [23] investigated the effect of the depth of screw insertion within the distal radius on fracture stability and found no significant difference between the three groups (bicortical vs. monocortical to the distal cortex vs. monocortical 75% to the distance of the distal cortex). Weninger et al. [24] compared three different screw configurations using a biomechanical synthetic model and again found no significant difference between the three groups (distal row only, two rows of screws: one group with parallel, the other with the proximal row inclined screws).

Several limitations of the study must be discussed. First, although the fracture model proposed here more closely resembles the in vivo situation, distal radius fractures can theoretically take on near infinite patterns. Still, as stated above, the distal fracture line was found to be well-defined and independent of fracture complexity, energy of the fall or age [19]. Therefore the authors believe this model to be representative of the majority of AO-23-A3 fractures. Second, the specimens were tested only in axial loading using a rapidly increasing cyclic loading protocol. The influence of other bending models (i.e. torsion, eccentric bending, combined axial compression and bending, etc.) and actual fatigue testing might provide additional information on the anchorage of the screws in the distal bone stock. However, dehydration of the specimens during long-term cyclic testing might affect the outcome. Third, specimen exposure time is a possibly limiting factor for any biomechanical test using fresh-frozen cadaveric bones. In this study, total exposure time for each specimen was below 24 h and the standardized preparation and testing protocol ensured a similar thawing-to-testing timing. Both parameters where shown to affect bone properties significantly [22]. Fourth, displacement was measured as actuator displacement, which must not necessarily reflect actual fragment displacement. As bending of the radius shaft was minimized by a short uncovered shaft, the authors believe actuator displacement to be sufficiently accurate. Fifth, force-displacement curves of three pairs showed no significant difference between the two fracture models. This was most probably due to early failure of the new osteotomy group specimens.

Conversely, the use of an established loading protocol [20] and paired fresh-frozen radii are strengths of the present study. All force-displacement curves showed a similar behavior for lower loads, which is indicative of similar plate placement and proves the reproducibility of the biomechanical setup. Moreover, the ultimate failure loads for the gold-standard model (1250 N ± 340.7 N) are in the range of previous studies [12,25,26]. Paired testing and alternation of intervention side ensured homogeneity within the two groups, which was confirmed by similar BMD and BMC values for each group. Overall, BMD and BMC were comparable to previous studies [27,28].

Overall, the above mentioned findings have implications for future biomechanical research, design of osteosynthesis devices and in vivo fracture treatment. Implementing this fracture model as the current gold standard fracture model will allow for better inter-study comparisons, independent of the loading protocol used. The new fracture model revealed greater displacement during axial loading than previously observed in biomechanical tests. This might have an impact on the material properties of novel osteosynthesis devices, as less displacement could result in fewer cases of secondary loss of reduction. Further research is needed to investigate the influence of the stiffness of osteosynthetic devices, secondary loss of reduction and screw cutting-through. Finally, the fact that the distal bone stock is significantly smaller than estimated in the old model underlines the need of polyaxial locking plates. As stated above, the screws inserted through the proximal screw row did fully engage in the bone stock for the gold standard fracture model but were only partially engaged in the smaller bone stock of the novel setup. Polyaxial locking systems are needed for adequate fracture reduction and retention.

## Conclusion

Distal radius fractures are one of the most common fractures, and biomechanical models are used to validate and develop novel treatment methods. Recent research suggests that the distal fracture fragment is smaller than observed in previous studies. Based on this data, an improved fracture model was developed. We were able to show that the biomechanical parameters assessed through biomechanical fracture models are sensitive to the position of the extra-articular comminuted fracture. The degree of sensitivity is dependent on the type of osteosynthetic device used. Consequently the fracture model introduced here should be used as the new gold standard for future research until more studies on fracture location have been done.

## Abbreviations

DRF: Distal radius fracture; mm: Millimeter; N: Newton; p: p-value; N/mm: Newton per millimeter; N*mm: Newton times millimeter; BMD: Bone mineral density; BMC: Bone mineral content; μCT: Micro Computer Tomography; MTS: Material testing system; N/s: Newton per second; h: Hours; SD: Standard deviation; mgHA/cm^2^: Milligram Hydroxyapatite per square centimeter; mg: Milligram; n: Number.

## Competing interests

Medartis Inc. kindly provided the sawbones and osteosynthetic material. The study was funded by a small research grant of the International Bone Research Association (IBRA, Basel, CH). The authors declare that they have no competing interests.

## Authors’ contributions

BSF: Idea, planning, specimen preparation, paper preparation. DAE: Planning, biomechanical testing, preparation of materials and methods and proofreading. WP: CT scans, proofreading. DM: Statistics. AA: Specimen preparation, CT scans, proofreading. TH: Specimen collection and preparation, proofreading. ZPK: Idea, planning, supervision, final proofreading. All authors read and approved the final manuscript.

## Pre-publication history

The pre-publication history for this paper can be accessed here:

http://www.biomedcentral.com/1471-2474/13/252/prepub

## References

[B1] Henry MH Distal radius fractures: current concepts J Hand Surg Am 2008 33 1215 1227 10.1016/j.jhsa.2008.07.013 18762124

[B2] Orbay JL Touhami A Current concepts in volar fixed-angle fixation of unstable distal radius fractures Clin Orthop Relat Res 2006 445 58 67 16505728 10.1097/01.blo.0000205891.96575.0f

[B3] Lozano-Calderón SA Souer S Mudgal C Jupiter JB Ring D Wrist mobilization following volar plate fixation of fractures of the distal part of the radius J Bone Joint Surg Am 2008 90 1297 1304 10.2106/JBJS.G.01368 18519324

[B4] Liporace FA Gupta S Jeong GK Stracher M Kummer F Egol KA Koval KJ A biomechanical comparison of a dorsal 3.5-mm T-plate and a volar fixed-angle plate in a model of dorsally unstable distal radius fractures J Orthop Trauma 2005 19 187 191 10.1097/00005131-200503000-00006 15758672

[B5] Liporace FA Kubiak EN Jeong GK Iesaka K Egol KA Koval KJ A biomechanical comparison of two volar locked plates in a dorsally unstable distal radius fracture model J Trauma 2006 61 668 672 10.1097/01.ta.0000234727.51894.7d 16967005

[B6] Blythe M Stoffel K Jarrett P Kuster M Volar versus dorsal locking plates with and without radial styloid locking plates for the fixation of dorsally comminuted distal radius fractures: A biomechanical study in cadavers J Hand Surg Am 2006 31 1587 1593 10.1016/j.jhsa.2006.09.011 17145377

[B7] Kandemir U Matityahu A Desai R Puttlitz C Does a volar locking plate provide equivalent stability as a dorsal nonlocking plate in a dorsally comminuted distal radius fracture?: a biomechanical study J Orthop Trauma 2008 22 605 610 10.1097/BOT.0b013e318186006f 18827589

[B8] Strauss EJ Banerjee D Kummer FJ Tejwani NC Evaluation of a novel, nonspanning external fixator for treatment of unstable extra-articular fractures of the distal radius: biomechanical comparison with a volar locking plate J Trauma 2008 64 975 981 10.1097/TA.0b013e3180eea9f0 18404064

[B9] Rausch S Klos K Stephan H Hoffmeier K Gras F Windolf M Gueorguiev B Hofmann GO Mückley T Evaluation of a polyaxial angle-stable volar plate in a distal radius C-fracture model - A biomechanical study Injury 2011 42 11 1248 1252 10.1016/j.injury.2010.12.005 21329924

[B10] Ekenstam F Hagert CG The distal radio ulnar joint. The influence of geometry and ligament on simulated Colles' fracture. An experimental study Scand J Plast Reconstr Surg 1985 19 27 31 10.3109/02844318509052862 4023640

[B11] Dunning CE Lindsay CS Bicknell RT Patterson SD Johnson JA King GJ Supplemental pinning improves the stability of external fixation in distal radius fractures during simulated finger and forearm motion J Hand Surg Am 1999 24 992 1000 10.1053/jhsu.1999.0992 10509278

[B12] Koh S Morris RP Patterson RM Kearney JP Buford WL Viegas SF Volar fixation for dorsally angulated extra-articular fractures of the distal radius: a biomechanical study J Hand Surg Am 2006 31 771 779 10.1016/j.jhsa.2006.02.015 16713841

[B13] Gondusky JS Carney J Erpenbach J Robertson C Mahar A Oka R Thompson M Mazurek M Biomechanical comparison of locking versus nonlocking volar and dorsal T-plates for fixation of dorsally comminuted distal radius fractures J Orthop Trauma 2011 25 44 50 10.1097/BOT.0b013e3181d7a3a6 21085029

[B14] Peine R Rikli DA Hoffmann R Duda G Regazzoni P Comparison of three different plating techniques for the dorsum of the distal radius: a biomechanical study J Hand Surg Am 2000 25 29 33 10.1053/jhsu.2000.jhsu025a0029 10642470

[B15] Cooper EO Segalman KA Parks BG Sharma KM Nguyen A Biomechanical stability of a volar locking-screw plate versus fragment-specific fixation in a distal radius fracture model Am J Orthop 2007 36 E46 E49 17703264

[B16] Rogge RD Adams BD Goel VK An analysis of bone stresses and fixation stability using a finite element model of simulated distal radius fractures J Hand Surg Am 2002 27 86 92 10.1053/jhsu.2002.29485 11810619

[B17] Augat P Reeb H Claes LE Prediction of fracture load at different skeletal sites by geometric properties of the cortical shell J Bone Miner Res 1996 11 1356 1363 8864911 10.1002/jbmr.5650110921

[B18] Augat P Iida H Jiang Y Diao E Genant HK Distal radius fractures: mechanisms of injury and strength prediction by bone mineral assessment J Orthop Res 1998 16 629 635 10.1002/jor.1100160517 9820289

[B19] Baumbach SF Schmidt R Varga P Heinz T Vécsei V Zysset PK Where is the distal fracture line location of dorsally displaced distal radius fractures? J Orthop Res 2011 29 489 494 10.1002/jor.21268 21337388

[B20] Varga P Baumbach S Pahr D Zysset PK Validation of an anatomy specific finite element model of Colles' fracture J Biomech 2009 42 1726 1731 10.1016/j.jbiomech.2009.04.017 19467661

[B21] Wolfram U Wilke H-J Zysset PK Damage accumulation in vertebral trabecular bone depends on loading mode and direction J Biomech 2011 44 1164 1169 10.1016/j.jbiomech.2011.01.018 21295781

[B22] Cartner JL Hartsell ZM Ricci WM Tornetta P Can We Trust Ex Vivo Mechanical Testing of Fresh-Frozen Cadaveric Specimens? The Effect of Postfreezing Delays J Orthop Trauma 2011 25 8 459 461 10.1097/BOT.0b013e318225b875 21738060

[B23] Greenberg JA Warden S Izadi KD The effect of screw length on fracture stability in volar locked plating of distal radius fractures Read at the AAOS Annual Meeting; 2010 March 10–14; New Orleans, LA

[B24] Weninger P Dall'ara E Drobetz H Nemec W Figl M Redl H Hertz H Zysset P Multidirectional volar fixed-angle plating using cancellous locking screws for distal radius fractures - Evaluation of three screw configurations in an extra-articular fracture model Wien Klin Wochenschr 2010 123 1–2 4 10 21165706 10.1007/s00508-010-1488-9

[B25] Mehling I Müller LP Delinsky K Mehler D Burkhart KJ Rommens PM Number and locations of screw fixation for volar fixed-angle plating of distal radius fractures: biomechanical study J Hand Surg Am 2010 35 885 891 10.1016/j.jhsa.2010.03.027 20513572

[B26] Krukhaug Y Gjerdet NR Lundberg OJ Lilleng PK Hove LM Different osteosyntheses for Colles' fracture: a mechanical study in 42 cadaver bones Acta Orthop 2009 80 239 244 10.3109/17453670902947440 19404810 PMC2823159

[B27] Engelke K Libanati C Liu Y Wang H Austin M Fuerst T Stampa B Timm W Genant HK Quantitative computed tomography (QCT) of the forearm using general purpose spiral whole-body CT scanners: accuracy, precision and comparison with dual-energy X-ray absorptiometry (DXA) Bone 2009 45 110 118 10.1016/j.bone.2009.03.669 19345291

[B28] Varga P Pahr DH Baumbach S Zysset PK HR-pQCT based FE analysis of the most distal radius section provides an improved prediction of Colles' fracture load in vitro Bone 2010 47 5 982 988 10.1016/j.bone.2010.08.002 20692389

